# The role of dopamine in risk taking: a specific look at Parkinson’s disease and gambling

**DOI:** 10.3389/fnbeh.2014.00196

**Published:** 2014-05-30

**Authors:** Crystal A. Clark, Alain Dagher

**Affiliations:** Montreal Neurological Institute, McGill UniversityMontreal, QC, Canada

**Keywords:** impulse control disorders, impulsivity, reward, loss aversion, insula, ventral striatum

## Abstract

An influential model suggests that dopamine signals the difference between predicted and experienced reward. In this way, dopamine can act as a learning signal that can shape behaviors to maximize rewards and avoid punishments. Dopamine is also thought to invigorate reward seeking behavior. Loss of dopamine signaling is the major abnormality in Parkinson’s disease. Dopamine agonists have been implicated in the occurrence of impulse control disorders in Parkinson’s disease patients, the most common being pathological gambling, compulsive sexual behavior, and compulsive buying. Recently, a number of functional imaging studies investigating impulse control disorders in Parkinson’s disease have been published. Here we review this literature, and attempt to place it within a decision-making framework in which potential gains and losses are evaluated to arrive at optimum choices. We also provide a hypothetical but still incomplete model on the effect of dopamine agonist treatment on these value and risk assessments. Two of the main brain structures thought to be involved in computing aspects of reward and loss are the ventral striatum (VStr) and the insula, both dopamine projection sites. Both structures are consistently implicated in functional brain imaging studies of pathological gambling in Parkinson’s disease.

## Gambling as a disorder of reward and punishment processing

Pathological gambling can be conceptualized as a disorder of reward and punishment processing, whereby the gambler selects an immediate but risky opportunity to obtain money over the larger, more probable opportunity to save money (Ochoa et al., 2013). Indeed, gambling is typically conceptualized as a disorder of impulsivity, in which decision-making is rash and relatively uninfluenced by future consequences. Pathological gamblers demonstrate increased impulsivity and increased delayed discounting on laboratory measures (Verdejo-Garcia et al., 2008). The coupling of increased reward seeking behavior with insensitivity to negative consequences may explain the persistence of gambling in the face of overall monetary losses (Vitaro et al., 1999; Petry, 2001b; Cavedini et al., 2002). This conceptual framework is similar to that used in drug addiction, where seeking immediate gains while minimizing potential risks is ubiquitous. Hallmarks of addiction include cravings or compulsions, a loss of control, and continued engagement in behaviors that maintain the addiction despite repeated negative consequences (American Psychiatric Association, 2000). Similarly, pathological gambling can be referred to as a behavioral addiction because it shares many common features with drug-addiction, such as compulsion and loss of control over one’s behavior, as well as continuation of the behavior in the face of negative consequences (Grant et al., 2006; Goodman, 2008). Pathological gamblers exhibit uncontrollable cravings, tolerance, habituation, and withdrawal symptoms, similar to those of drug addicts (Wray and Dickerson, 1981; Castellani and Rugle, 1995; Duvarci and Varan, 2000; Potenza et al., 2003). Moreover, both pathological gambling and substance abuse are associated with the same specific personality traits, namely sensation seeking and impulsivity (Zuckerman and Neeb, 1979; Castellani and Rugle, 1995), which index heightened arousal to potential rewards and reduced self-control and inhibitory function. The high comorbidity between substance dependence (drugs and alcohol) and pathological gambling (Petry, 2001a; Petry et al., 2005), and evidence for common genetic factors, point to the two disorders having overlapping etiologies (Slutske et al., 2000; Goodman, 2008).

**Figure 1:**
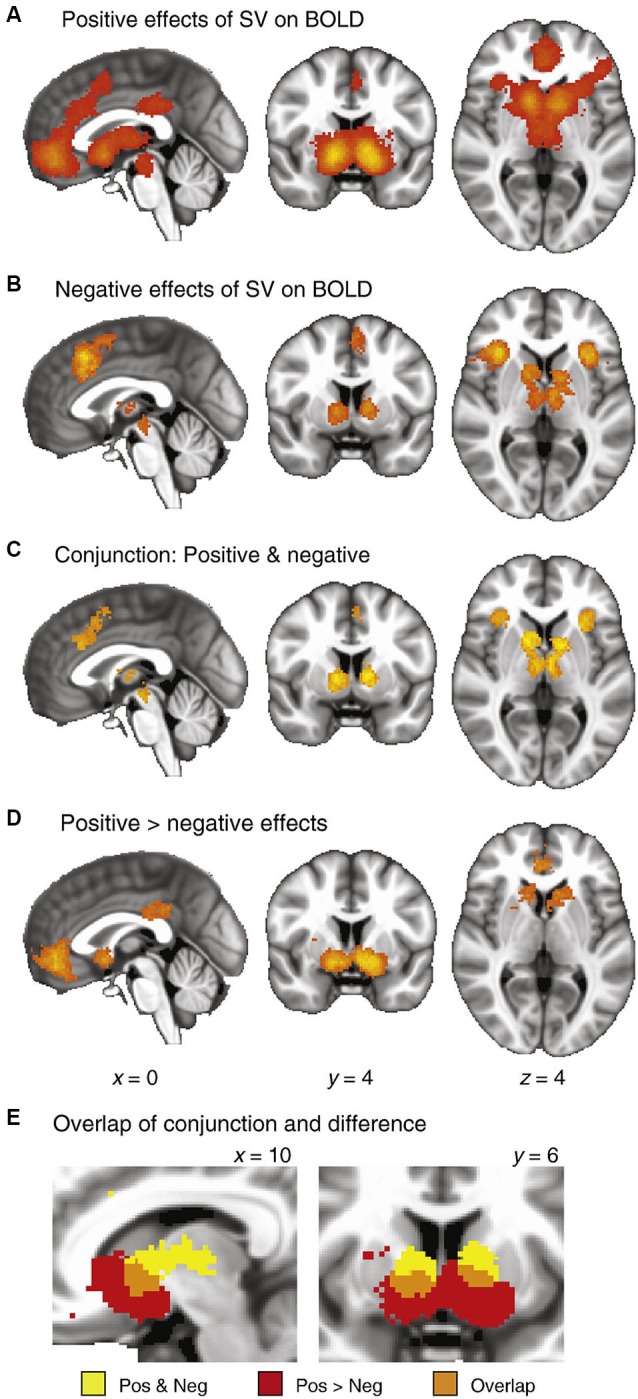
**Meta-analysis of fMRI studies of value (taken from Bartra et al., 2013)**. The authors extracted peak coordinates of activation from 206 published fMRI studies that investigated value computations. **(A)** Significant clustering of positive responses. **(B)** Significant clustering of negative responses. **(C)** Conjunction maps, showing regions with significant clustering for both positive and negative responses. **(D)** Results of a between-category comparison, showing regions with significantly greater clustering for positive than negative effects. **(E)** Detail of the striatum, illustrating overlap between the conjunction map (**Panel C**) and the difference map (**Panel D**). These data demonstrate the relative response of anterior insula, striatum and ventromedial PFC to positive and negative value.

One useful model views reward and punishment learning as inherent components in the decision-making process. Decision-making can be broken down to the weighing of the probability and value of reward against potential costs (e.g., negative consequences). Other factors such as outcome ambiguity and variance (sometimes referred to as risk) also affect individual choices (Huettel et al., 2006), but here we will only consider potential gains and losses as determinants of decision-making while gambling. We will also take “risk” to mean the potential loss attached to any choice. Risk, as so defined, increases with the magnitude and probability of potential losses. In fact, risk-taking may be seen as an indicator of the balance existing between computations of potential gains and losses. Two of the main brain structures thought to be involved in these computations are the ventral striatum (VStr) and the insula, both dopamine projection sites. Both have been linked to computations of value, with the VStr being especially responsive to reward prediction error (RPE), encoding gain anticipation positively and loss anticipation negatively (Rutledge et al., 2010; Bartra et al., 2013), and the insula responding predominantly to losses and loss anticipation in some studies (Knutson and Greer, 2008) or to both positive and negative outcomes in others (Campbell-Meiklejohn et al., 2008; Rutledge et al., 2010). Bartra et al.’s meta-analysis (Figure 1) suggests that the insula encodes arousal or salience as opposed to value, as it responds positively to both gains and losses. This meta-analysis also raises the possibility of a greater role for the insula in the assessment of risk and losses than gains (compare panels A and B in Figure 1). Alteration of the balance between these gain and loss anticipation systems may underlie the inappropriate choice behaviors that occur in disorders such as addiction, gambling and impulse control disorders.

Recent research suggests that differences in brain function, structure, and biochemistry are present in those who develop gambling problems, with dopamine being a common etiological factor. Imaging studies have demonstrated an increase in mesolimbic dopamine release during gambling tasks in healthy subjects (Thut et al., 1997; Zald et al., 2004; Hakyemez et al., 2008). However it should be noted that unpredictable reward tasks have the ability to cause a suppression and enhancement of dopamine transmission in different regions of the striatum (Zald et al., 2004; Hakyemez et al., 2008). Earlier research on pathological gamblers suggested altered dopaminergic and noradrenergic systems, as found through a decrease in concentration of dopamine and an increase in cerebrospinal fluid levels of 3,4-dihydroxyphenyl-acetic acid and homovanilic acid (Bergh et al., 1997). Pathological gamblers have also been reported to have higher cerebrospinal fluid levels of 3-methoxy-4-hydroxyphenylglycol, a major metabolite of norepinephrine, as well as significantly greater urinary outputs of norepinephrine in comparison to controls (Roy et al., 1988), indicative of a functional disturbance of the noradrenergic system. In addition there is evidence that genetic polymorphisms affecting dopaminergic neurotransmission act as risk factors for problem gambling (Lobo and Kennedy, 2006).

## Dopamine in reinforcement

Considerable evidence from animal studies, implicating dopamine in behavioral reinforcement, provides a neurobiological substrate that could encompass processing of natural rewards, such as food and sex, as well as drugs of abuse and pathological gambling (Di Chiara and Imperato, 1988; Wise and Rompre, 1989; Wise, 1996, 2013). The observations of Schultz and others (Schultz et al., 1998; Schultz, 2002) confirmed a role for dopamine neurons in response to rewards; however the current model of dopamine signaling can be traced to a seminal paper by Montague, Dayan and Schultz (Schultz et al., 1997), where it was argued that the firing pattern of dopamine neurons did not signal reward *per se*, but a RPE signal, similar to those used in machine learning. This finding, along with evidence that dopamine could modulate synaptic plasticity (Calabresi et al., 2007; Surmeier et al., 2010) led to the theory that dopamine acts as a learning (or reinforcement) signal that shapes future motivated behavior. Subsequent research has shown that dopamine may also encode predictions about upcoming rewards and reward rate, thus acting as a value signal in the mesocortical and mesolimbic dopaminergic pathways (Montague and Berns, 2002).

**Figure 2:**
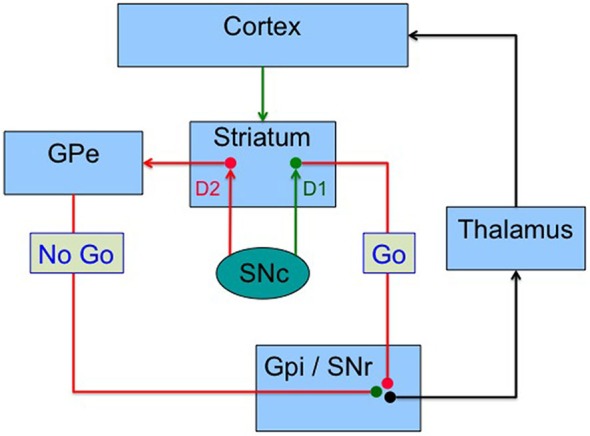
**Basal ganglia model.** A possible model whereby basal ganglia compute the utility of gains and losses via two segregated pathways in the corticostriato-thalamocortical circuit. Striatal output neurons of the direct pathway express D1 receptors and project to the internal globus pallidus (GPi) and the substantia nigra pars reticulata (SNr), and has an action selection effect on cerebral cortex. Striatal output neurons in the indirect pathway express D2 receptors and reduce the tonic inhibition of the external globus pallidus (GPe) on the GPi/SNr, which leads to action inhibition in the cortex. D1 receptors respond mainly to phasic (high concentration) dopamine signaling due to their low affinity for dopamine. D2 receptors have high affinity for dopamine and respond to lower tonic dopamine levels. Excitatory projections in green, inhibitory in red.

The main projection site of dopamine neurons is the striatum, whose connectivity to frontal, limbic and insular cortex, provides a mechanism whereby dopamine can act as a prediction error signal driving both “Go” learning, which relates to actions with positive outcomes, and “No Go” or avoidance learning, which relates to actions that lead to punishment or an absence of reward. First, dopamine signaling operates in two modes (Grace, 2000): slow constant release of dopamine regulates tonic levels, which mostly signal via dopamine D_2_ receptors on striatal medium spiny neurons; phasic bursts of dopamine firing lead to large increases in synaptic dopamine which signal via both the D_1_ and D_2_ receptor systems. D_1_ receptors have low affinity for dopamine (Marcellino et al., 2012) and only respond to large increases in synaptic dopamine released during phasic dopamine neuron bursts that reflect positive RPEs, supporting learning to approach rewarding stimuli (Frank, 2005). Dopamine D_2_ receptors, on the other hand, have a higher affinity for dopamine, allowing them to respond to tonic dopamine signaling, and to detect transient reductions in tonic dopamine levels that follow pauses in dopamine neuron firing during negative RPEs. This facilitates learning to avoid negative outcomes (Frank, 2005). The cortico-striatal system can be divided into a direct and an indirect pathway (Figure 2), which have opposite effects on the thalamus and hence cortex (Albin et al., 1989). In the dorsal striatum, receptors are segregated, with the D_1_ receptors within the direct pathway, related to action selection, while the D_2_ receptors control response inhibition within the indirect pathway (Mink, 1996). This separation allows dopamine to drive both reward (increases in dopamine signaling a better outcome than expected) and punishment (reductions in tonic dopamine indicated a worse outcome than expected). Frank proposed a model in which phasic dopamine bursts following rewards promote positive reinforcement while reductions in tonic dopamine levels lead to negative reinforcement, each controlled by the D_1_/direct pathway and the D_2_/indirect pathway, respectively (Cohen and Frank, 2009). This computational model suggests that the RPE dopamine signal promotes learning from positive outcomes via stimulation of D_1_ receptors, whereas learning to avoid negative outcomes is mediated via disinhibition of indirect pathway striatal neurons secondary to a reduction of D_2_ receptor stimulation during dopamine pauses (Cohen and Frank, 2009). A negative outcome (punishment or lack of an expected reward) leads to pause in the firing of dopamine neurons, which then leads to a transient reduction in tonic dopamine. It should also be noted that D_2_ receptor stimulation reduces excitability of neurons in the indirect pathway (Hernandez-Lopez et al., 2000), therefore, reductions in D_2_ receptor signaling have the effect of activating the inhibitory “No Go” pathway. This allows for bidirectional positive and negative reinforcement signaling by dopamine neurons. Support for this model has been provided by numerous experiments. Parkinson’s disease patients show enhanced positive learning when on their medications, but improved negative learning while off medication (Frank et al., 2004). Pharmacological manipulations also support the model (Frank and O’Reilly, 2006; Pizzagalli et al., 2008). The striatal release of dopamine is linked to associative learning and habit formation via control of corticostriatal synaptic plasticity, which is affected in an opposite manner by D_1_ and D_2_ signaling (Shen et al., 2008). D_1_ dopamine receptor signaling promotes long-term potentiation (Reynolds et al., 2001; Calabresi et al., 2007), whereas D_2_ receptor signaling promotes long-term depression (Gerdeman et al., 2002; Kreitzer and Malenka, 2007). Note that this model has been tested most thoroughly at the level of the striatum. Multivariate analysis of fMRI data shows that reinforcement and punishment signals are ubiquitous in the brain, most notably in the entire frontal cortex and striatum (Vickery et al., 2011). Less is known about the information signaled by dopamine projections to brain areas other than the striatum, such as frontal cortex, insula, hippocampus and amygdala, or how the RPE signal is used by these areas.

## Striatum and monetary reward

In human functional neuroimaging studies, changes in brain activation have been demonstrated consistently in response to monetary rewards (Thut et al., 1997; Elliott et al., 2000; Knutson et al., 2000; Breiter et al., 2001; O’Doherty et al., 2007). Further, studies have teased apart the different brain areas involved in the various components of monetary reward, such as anticipation, feedback, winning and losing. There seems to be a specialization within dopamine projection sites in relation to monetary reward: anticipation of monetary reward increases activation in the VStr, which includes the nucleus accumbens, while rewarding outcomes increase activation in the ventral medial prefrontal cortex, dorsal striatum, and posterior cingulate, with deactivation in the aforementioned regions during reward omission (Elliott et al., 2000; Breiter et al., 2001; Knutson et al., 2001b; Tricomi et al., 2004). Neuroimaging experiments in humans suggest that VStr activity strongly correlates with expected value, as well as magnitude and probability (Breiter et al., 2001; Knutson et al., 2001a, 2005; Abler et al., 2006; Yacubian et al., 2006; Rolls et al., 2008). Work by D’Ardenne et al. (2008) supports a role for the mesolimbic dopamine system in monetary RPE signaling. Activation of the ventral tegmental area, the origin of the mesolimbic dopamine circuit, reflected positive RPEs, whereas the VStr encoded positive and negative RPEs. Similarly, Tom et al. (2007) showed that VStr activity reflected potential monetary gains and losses bidirectionally. This study also demonstrated that these neural signals reflected individual variations in loss aversion, the tendency for losses to be more impactful than potential gains. Finally, the influential actor-critic model (Sutton and Barto, 1998) proposes that the VStr uses prediction errors to update information about expected future rewards while the dorsal striatum uses this same prediction error signal to encode information about actions that are likely to lead to reward. This distinction has found support from fMRI experiments (O’Doherty et al., 2004; Kahnt et al., 2009). Interestingly, the ability to update behavior in response to RPE was shown to correlate with functional connectivity between dorsal striatum and dopaminergic midbrain (Kahnt et al., 2009). The imaging studies mentioned here support the theory of dopamine as a RPE signal, at least in its striatal projection.

## Insula and risk

The insula is frequently activated in functional neuroimaging experiments (Duncan and Owen, 2000; Yarkoni et al., 2011). Functionally it can be divided into three distinct subregions: a ventroanterior region associated with chemosensory (Pritchard et al., 1999) and socio-emotional processing (Sanfey et al., 2003; Chang and Sanfey, 2009), a dorsoanterior region associated with higher cognitive processing (Eckert et al., 2009), and a posterior region associated with pain and sensorimotor processing (Craig, 2002; Wager et al., 2004). Different functional insular areas project to different striatal targets: the VStr receives insular projections primarily related to food and reward, whereas the dorsolateral striatum receives insular inputs related to somatosensation (Chikama et al., 1997).

The insular cortex is involved in decision-making processes that involve uncertain risk and reward. Specifically, fMRI studies have reported insular cortex involvement in risk-averse decisions (Kuhnen and Knutson, 2005), risk avoidance and the representation of loss prediction (Paulus et al., 2003), monetary uncertainty (Critchley et al., 2001), and encoding a risk prediction error (Preuschoff et al., 2008). Patients with insular cortex damage place higher wagers in comparison with healthy participants and their betting is less sensitive to the odds of winning, with high wagers even at unfavorable odds (Clark et al., 2008). Other research suggests that optimum decisions involving risk depend on the integrity of the insular cortex, showing that insula lesion patients have altered decision-making involving both risky gains and risky losses (Weller et al., 2009) (However see Christopoulos et al., 2009). Specifically, insula damage was associated with a relative insensitivity to expected value differences between choices. Previous research has shown that there is a dissociation between insula and VStr, with VStr activation preceding risk-seeking choices, and anterior insula activation predicting risk-averse choices (Kuhnen and Knutson, 2005) suggesting that the VStr represents gain prediction (Knutson et al., 2001a), while anterior insula represents loss prediction (Paulus et al., 2003). While imaging studies also demonstrate a more general role of the anterior insula in signaling the valence (positive or negative) of potential rewards (Litt et al., 2011; Bartra et al., 2013) the lesion data argue that the anterior insular cortex has a role in risk evaluation, specifically in making risk-averse decisions. Indeed, in healthy subjects, the insula is part of a value network that appears to track potential losses in a way that correlates with individual loss aversion level (Canessa et al., 2013). It is possible that an imbalance between prefrontal-striatal circuitry and insular-striatal circuitry may lead to suboptimal choices when weighing potential gains and losses, as observed in pathological gamblers (Petry, 2001a; Goudriaan et al., 2005).

## Pathological gambling among patients with Parkinson’s disease

Pathological gambling was first reported in the context of Parkinson’s disease and dopamine replacement therapy in 2000 (Molina et al., 2000). The lifetime prevalence of pathological gambling in the general public is approximately 0.9 to 2.5% (Shaffer et al., 1999). In Parkinson’s disease, the prevalence rates are higher, from 1.7 to 6.1% (Ambermoon et al., 2011; Callesen et al., 2013). The risk factors associated with the occurrence of pathological gambling in Parkinson’s disease are young age of Parkinson’s disease onset, a personal or family history of drug or alcohol abuse, depression, and relatively high impulsivity and novelty seeking personality scores (Voon et al., 2007b). Interestingly, these are similar to the risk factors for drug addiction and pathological gambling in the general population. Also, there have been reports of addiction to L-dopa in certain patients (e.g., Giovannoni et al., 2000), a phenomenon that had already been noted in the 1980s. It was perhaps initially surprising to find that Parkinson’s disease patients can become addicted to their own medication or develop behavioral addictions because they were thought to not possess the personality type typical of addicted individuals. They are generally described as industrious, punctual, inflexible, cautious, rigid, introverted, slow-tempered, with lack of impulsiveness and novelty seeking, and they have low lifetime risks for cigarette smoking, coffee drinking, and alcohol use predating Parkinson’s disease onset (Menza et al., 1993; Menza, 2000).

Dopamine replacement therapy has been implicated in the development of pathological gambling in Parkinson’s disease (Gschwandtner et al., 2001; Dodd et al., 2005) and a remission or reduction of pathological gambling is typically noted after reduction or cessation of dopamine agonist medication (Gschwandtner et al., 2001; Dodd et al., 2005). A broader set of behavioral addictions termed impulse control disorders, including but not limited to pathological gambling, compulsive sexual behavior, and compulsive buying, have been reported in association with dopamine replacement therapy (Weintraub et al., 2006; Voon et al., 2007a; Dagher and Robbins, 2009). Dopamine agonists (pramipexole, ropinirole and pergolide) appear to pose a greater risk than L-Dopa monotherapy (Seedat et al., 2000; Dodd et al., 2005; Pontone et al., 2006). Reducing the dopamine agonist and increasing L-Dopa to achieve same motor response abolished pathological gambling in affected individuals (Mamikonyan et al., 2008), while a cross-sectional study of over 3000 Parkinson’s disease patients found that taking a dopamine agonist increased the odds of developing an impulse control disorder by 2.72 (Weintraub et al., 2010). Finally, these side-effects of dopamine agonist therapy have been recently noted in other diseases, such as restless leg syndrome, fibromyalgia and prolactinomas (Davie, 2007; Driver-Dunckley et al., 2007; Quickfall and Suchowersky, 2007; Tippmann-Peikert et al., 2007; Falhammar and Yarker, 2009; Holman, 2009). It should be noted however that some studies have reported behavioral addictions and/or impulsivity and compulsivity in association with high-dose L-Dopa monotherapy (Molina et al., 2000), deep brain stimulation for Parkinson’s disease (Smeding et al., 2007), and in drug naïve Parkinson’s disease patients (Antonini et al., 2011), all in the absence of dopamine agonists. Nonetheless, the clinical evidence overwhelmingly supports the theory that dopamine agonism at the D_2_ receptor family is sufficient to cause impulse control disorders.

## Brain imaging studies

### Neurotransmitter imaging

Positron emission tomography (PET) imaging allows for changes in endogenous levels of dopamine to be inferred from changes in the binding of the [^11^C]raclopride to the dopamine D_2_ receptors. The first [^11^C]raclopride PET study in this area was on Parkinson’s patients with dopamine dysregulation syndrome. Dopamine dysregulation syndrome is characterized by the compulsive taking of dopaminergic drugs, which is often comorbid with impulse control disorders (Lawrence et al., 2003). Patients with dopamine dysregulation syndrome exhibited enhanced L-Dopa induced VStr dopamine release compared to similarly treated Parkinson’s disease patients not compulsively taking dopaminergic drugs (Evans et al., 2006). This was the first study to provide evidence for sensitization of mesolimbic dopamine circuitry in Parkinson’s disease patients prone to compulsive drug use. Subsequent studies have supported a relative hyperdopaminergic state in Parkinson’s disease patients with pathological gambling. Three studies mapping the concentration of dopamine reuptake transporters (DAT) have shown reduced levels in the VStr of Parkinson’s disease patients with impulse control disorders compared to unaffected patients (Cilia et al., 2010; Lee et al., 2014; Voon et al., 2014). Unfortunately the finding is non-specific, as reduced DAT concentration can index either reduced nerve terminals (and reduced dopamine signaling) or reduced DAT expression (and therefore increased tonic dopamine levels). Supporting the latter hypothesis, impulse control patients demonstrate reduced [^11^C]raclopride binding in the VStr compared to Parkinson’s controls (Steeves et al., 2009), which is also consistent with elevated tonic dopamine in this group. Note, however that this result failed to be replicated in a similar study (O’Sullivan et al., 2011).

However, these two [^11^C]raclopride PET studies reported a greater reduction of VStr binding potential (an index of dopamine release) during gambling (Steeves et al., 2009) and following reward-related cue exposure (images of food, money, sex) compared to neutral cues (O’Sullivan et al., 2011) in Parkinson’s disease patients with impulse control disorders compared to unaffected patients. This suggests an increased responsiveness of striatal reward circuitry to gambling and reward-related cues in those patients with impulse control disorders. In O’Sullivan et al. (2011) dopamine release was only detected in the VStr and only when subjects received a dose of oral L-Dopa just prior to scanning, consistent with post-mortem data in Parkinson’s disease showing that brain dopamine levels are much lower in dorsal than VStr (Kish et al., 1988). These results are therefore consistent with the sensitization hypothesis proposed by Evans et al. (2006). More recently it was reported that Parkinson’s disease patients with pathological gambling have a reduced concentration of dopamine autoreceptors in the midbrain (Ray et al., 2012), which is known to correlate with elevated dopaminergic responsivity and increased impulsivity (Buckholtz et al., 2010). Finally, in Parkinson’s disease patients, dopamine synthesis capacity, as measured by [^18^F]DOPA PET, correlates with a personality measure of disinhibition, itself a risk factor for pathological gambling and other addictions (Lawrence et al., 2013). In summary, PET studies provide converging evidence of heightened dopaminergic tone and increased dopamine response to reward cues as the underlying vulnerability in Parkinson’s disease patients who develop pathological gambling during dopamine agonist treatment.

### Functional magnetic resonance imaging

Parkinson’s disease patients with pathological gambling show enhanced hemodynamic responses to gambling-related visual cues in the bilateral anterior cingulate cortex, left VStr, right precuneus and medial prefrontal cortex (Frosini et al., 2010). This is in line with similar experiments in pathological gambling without Parkinson’s disease (Crockford et al., 2005; Ko et al., 2009) and drug addiction (Wexler et al., 2001), supporting the view that impulse control disorders in Parkinson’s disease may be conceptualized as behavioral addictions.

Parkinson’s disease patients with an impulse control disorder show diminished BOLD activity in the right VStr during risk taking and significantly reduced resting cerebral blood flow in the right VStr compared to their healthy disease counterparts (Rao et al., 2010). Similarly, it was found that Parkinson’s disease patients with impulse control disorders showed a bias toward risky gambles compared to control patients, and that dopamine agonists enhanced risk taking while decreasing VStr activity (Voon et al., 2011). The authors suggested that dopamine agonists may decouple brain activity from risk information in vulnerable patients, thus favoring risky choices. Another fMRI study reported that, relative to Parkinson’s controls, impulse control disorder Parkinson’s patients had decreased anterior insular and orbitofrontal cortex RPE signals. They also showed that dopamine agonists increased the rate of learning from gain outcomes, and increased striatal RPE activity, suggesting that dopamine agonists may skew neural activity to encode “better than expected” outcomes in Parkinson’s disease patients susceptible to impulse control disorders (Voon et al., 2010).

While differences in striatal dopamine signaling may distinguish Parkinson’s disease patients who do and do not develop pathological gambling, the mechanism of action by which dopamine agonists change risk assessment remains unclear. Dopamine agonists change the way in which the brains of healthy individuals respond to the anticipation and feedback of rewards. During reward feedback, administration of a single dose of pramipexole to healthy adults caused decreased VStr activity in a lottery game (Riba et al., 2008). Similarly, there was reduced VStr activation when Parkinson’s patients received a dose of L-Dopa compared to placebo (Cools et al., 2007). This pattern of hypoactivation is reminiscent of that found in pathological gamblers without Parkinson’s disease (Reuter et al., 2005): during a simulated gambling task, pathological gamblers showed decreased activation with respect to controls in the ventromedial prefrontal cortex and the VStr. Severity of gambling was negatively correlated with the BOLD effect in the VStr and ventromedial prefrontal cortex, suggesting that hypoactivity is a predictor of gambling severity. As noted above, impulse control disorder Parkinson’s patients were found to have diminished resting perfusion as well as diminished BOLD activity during risk taking in the VStr compared to Parkinson’s controls (Rao et al., 2010). These studies suggest that dopamine agonists cause individuals to seek rewards and make risky choices (Riba et al., 2008), in the face of suppressed VStr response to rewards.

It should be noted however that reduced VStr activation in fMRI experiments does not necessarily indicate reduced dopaminergic signaling. There is evidence to support relatively spared mesolimbic dopamine signaling as the risk factor for pathological gambling in Parkinson’s disease. First, the repeated taking of a dopaminergic medication for the treatment of Parkinson’s disease could lead to sensitization of dopamine signaling. VStr sensitization has been shown following repeated amphetamine administration in humans (Boileau et al., 2006). Moreover, in Parkinson’s disease the ventral portion of striatum is relatively spared by the disease compared to the dorsal areas (Kish et al., 1988), and thus dopamine replacement therapy, while correcting the dopamine deficiency in the dorsal striatum to normal levels, has the potential to raise dopamine levels in the VStr circuit to higher than optimal levels (Cools et al., 2007). This “overdose” theory was first proposed by Gotham et al. (1988) to explain the fact that L-Dopa administration to Parkinson’s disease patients, while improving some cognitive deficits, could also cause specific impairments in other fronto-striatal cognitive tasks. In the case of impulse control disorders, we propose that excessive dopaminergic stimulation in the VStr obscures the dips in dopamine signaling related to negative prediction errors.

The insula has also been implicated in imaging studies of pathological gambling in Parkinson’s disease. In an fMRI study, Ye et al. (2010) found that during the anticipation of monetary rewards, a single dose of pramipexole (compared to placebo) increased the activity of the VStr, enhanced the interaction between the VStr and the anterior insula, but weakened the interaction between the VStr and the prefrontal cortex, leading to increased impulsivity. Cilia et al. (2008) found Parkinson’s patients with pathological gambling showed resting over-activity in brain areas in the mesocorticolimbic network, including the insula. In an fMRI study, relative to Parkinson’s controls, impulse control disorder patients had decreased anterior insular and orbitofrontal cortex activity (van Eimeren et al., 2009; Voon et al., 2010). Finally, in a study of Parkinson’s disease patients with and without hypersexuality, a single dose of L-Dopa abolished the normal insular deactivation seen in response to erotic pictures, only in the hypersexual patients (Politis et al., 2013). Taken together these results may suggest an imbalance between the prefrontal-striatum connectivity and insula-striatum connectivity, favoring the influence of potential gains over that of potential risks (losses) in decision-making.

## Risk taking and loss aversion

An influential framework for studying risky decision making is prospect theory, developed by Kahneman and Tversky (1979). A key finding of their work is loss aversion, a tendency for losses to loom larger than potential gains, and for individuals to typically forego risky choices when less valuable safer alternatives exist. For example most people will reject the offer of a coin flip unless the potential gain is considerably larger than the potential loss. Impulsiveness, at least in a gambling context, can be characterized as a reversal of loss aversion, and an over-weighing of potential rewards relative to losses. It remains to be seen whether loss aversion results from asymmetrical weighting of gains and losses along a single value axis (Tom et al., 2007), or from a competitive interaction between separate systems for gains and losses (Kuhnen and Knutson, 2005; De Martino et al., 2010). Possibly, both models are correct: recent fMRI evidence (Canessa et al., 2013) shows bidirectional responses to losses and gains in the VStr and ventromedial prefrontal cortex (positive for gains) and the amygdala and insula (positive for losses). In both cases, there is greater activation to potential losses, correlating with individual loss aversion measured using prospect theory (Kahneman and Tversky, 1979). However, there are also brain regions that respond uniquely to potential losses, namely the right insula and the amygdala, once again reflecting individual variation in loss aversion (Canessa et al., 2013). In sum, a network of regions centered on VStr, insula and amygdala seems to compute gain and loss anticipation in a way that typically results in loss aversion. Interestingly these structures, along with dorsal anterior cingulate, form an intrinsic connectivity network as identified by resting state fMRI. This network is thought to be involved in detecting and processing emotionally salient events (Seeley et al., 2007).

Loss aversion can be explained on an emotional basis, with both potential gains and losses influencing behavior via different emotions (Loewenstein et al., 2001), namely motivation on the gain side and anxiety for losses. Such a model might tie the former to the nucleus accumbens and the latter to the amygdala and insula. In either case, it is conceivable that individuals who are relatively less loss averse may also be at risk for impulsive behaviors such as drug addiction and gambling, due to relative under valuation of losses, although surprisingly this has yet to be formally tested.

There is some evidence implicating the striatum in reversal of normal loss aversion in pathological gamblers. Loss of striatal dopamine neurons in Parkinson’s disease is associated with reduced risk-taking behavior compared to control subjects (Brand et al., 2004; Labudda et al., 2010), while chronic administration of dopamine agonists, especially in high doses, reverses this tendency and promotes risky behavior and impulsivity (Dagher and Robbins, 2009). In the healthy brain, acute administration of D_2_ dopamine agonists may also cause an increase in risky choices in humans (Riba et al., 2008) and rats (St Onge and Floresco, 2009). Acute D_2_/D_3_ receptor stimulation has been found to produce complex changes in the value of losses judged worth chasing (chasing being the continued gambling to recover losses) (Campbell-Meiklejohn et al., 2011). Taken together, this suggests dopamine, acting on the striatum and possibly other mesolimbic structures, may modulate loss aversion. Two studies in Parkinson’s disease patients not affected by impulse control disorders found that a single dose of the dopamine agonist pramipexole reduced loss prediction error coding in the orbitofrontal cortex in one case (van Eimeren et al., 2009) and the orbitofrontal cortex and insula in the other (Voon et al., 2010). In sum, tonic dopamine activity appears to reduce loss prediction signaling, and may therefore reduce loss aversion.

**Figure 3:**
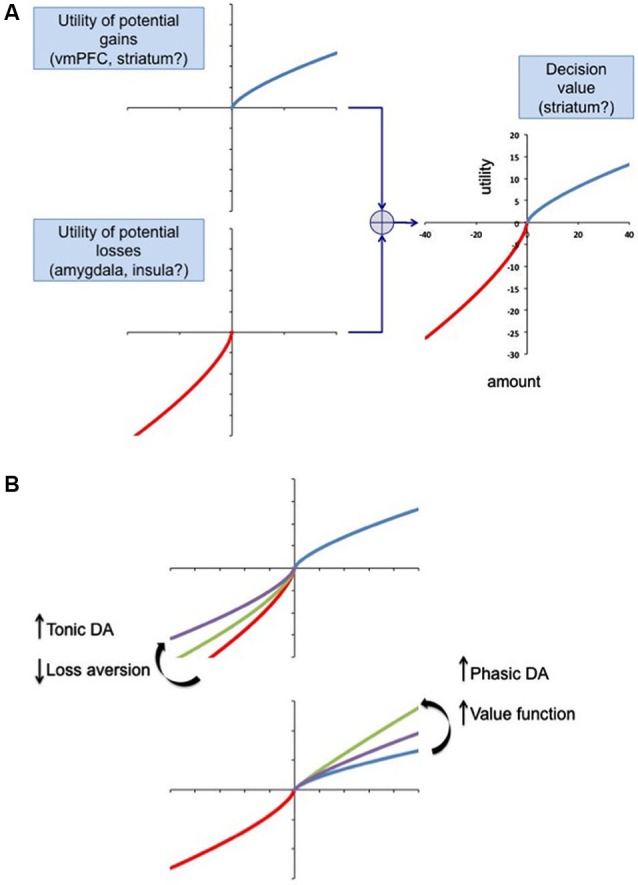
**A model of decision-making based on prospect theory. (A)** The utility of potential gains and losses is given by the following equation: *u*(*x*) = (*x*)^*α*^ for potential gains and *u*(*x*) = −*λ* · (−*x*)^*β*^ for losses (Kahneman and Tversky, 1979). When the loss aversion parameter *λ* is greater than 1 the function is steeper in the loss domain, implying loss aversion. In this model the utility of gains and losses is computed by different neural networks and combined at some point. We list regions that may be implicated in the calculation. **(B)** Dopamine may influence the shape of the utility function for gains and losses, by affecting any of the parameters *α*, *β* or *λ* to regulate the degree of loss aversion. Tonic and phasic dopamine may modulate gain and loss calculation via the direct and indirect basal ganglia pathways (Figure 2). The balance of tonic and phasic dopamine signaling could regulate the balance between action selection and inhibition, regulating the current level of loss aversion.

We propose a general framework based on prospect theory, in which the anticipation of potential losses and rewards is computed, possibly in separate brain regions initially, and integrated to compute a decision value (Figure 3). We speculate that gain anticipation might be computed in the ventral medial prefrontal cortex, based on numerous imaging studies implicating this area in computation of value (Kable and Glimcher, 2007; Plassmann et al., 2007; Bartra et al., 2013). As reviewed above, the amygdala and insula may be involved in computing loss anticipation. A possible site for the final computation of value, at least for the purpose of updating choices and action plans, is the striatum, which has fairly direct access to brain regions involved in action planning (van der Meer et al., 2012). The striatum has inherent roles in both response-reward associations (dorsal striatum) (Alexander and Crutcher, 1990) and creating stimulus-reward contingencies (VStr), which afford it the unique opportunity for computation of value (Packard and Knowlton, 2002). Striatal value signals can promote reinforcement processes leading to the updating of future actions, strategies and habits, mediated by the dorsal striatum, while also driving appetitive reward seeking behavior via the VStr. For a review of the role of the striatum in value coding see Knutson et al. (2008); Bartra et al. (2013). The balance between gain and loss evaluation systems may be modulated at least in part by dopamine. We propose a model in which tonic dopamine, acting via the indirect basal ganglia pathway (Figure 2) regulates inhibitory control manifesting as loss aversion. Here lower levels of tonic dopamine would be associated with increased loss aversion. Conversely, phasic dopamine, acting via the direct pathway, would increase the value of gains. This is based on the finding that young healthy subjects given a single dose of the dopamine agonist cabergoline show reduced learning in response to gains (positive feedback), due presumably to a presynaptic effect (in low doses, cabergoline, a D_2_ agonist, reduces phasic dopamine neuron firing via actions on the high affinity D_2_ autoreceptor, located pre-synaptically on dopamine neurons) (Frank and O’Reilly, 2006). Conversely, haloperidol, a D_2_ antagonist, increased learning from gains, probably due to its ability to enhance phasic dopamine firing. With respect to Parkinson’s disease, if a patient has an individual vulnerability to undervalue losses, then dopamine agonist therapy, which tonically stimulates D_2_ receptors and blocks sensing of the phasic dopamine dips associated with negative rewards, (Frank et al., 2004, 2007), could result in even lower loss aversion. One interpretation is that the intensity of phasic activity sets the gain on the value of potential rewards, while the tonic stimulation of D_2_ receptors blocks the negative feedback associated with losses.

Parkinson’s disease patients show enhanced positive learning when on dopaminergic medications, and improved negative learning while off medication, compared to age-matched controls (Frank et al., 2004). Treatment with dopamine D_2_ agonists is now accepted as the cause of impulse control disorders in Parkinson’s disease, in which problem gambling is phase locked to medication use. In the model proposed here, D_2_ stimulation would reduce loss aversion via the indirect corticostriatal pathway. We suggest that under D_2_ agonist treatment, these patients have a tendency to undervalue losses and be more risk seeking. This is consistent with the observation that Parkinson’s disease patients’ deficits in risky decision making is dominated by impaired ability to use negative feedback (Labudda et al., 2010). The effect on gain, risk, and loss processing of dopamine signaling in other parts of the mesolimbic and mesocortical system, notably the vmPFC, OFC, insula and amygdala, remains to be investigated in greater depth.

Loss tolerance profile may also be affected by norepinephrine signaling. In healthy volunteers, a single dose of the centrally acting beta blocker propranolol reduced the perceived magnitude of losses (Rogers et al., 2004) and normal variations in norepinephrine reuptake transporter in the thalamus, as assessed by PET, correlate with loss aversion (Takahashi et al., 2013). An explanation for this is that norepinephrine increases the arousal response to potential losses, and low norepinephrine signaling may therefore reduce loss aversion. While norepinephrine neurons are also affected in Parkinson’s disease, their role in the motivational and impulsive aspects of the disease have yet to be investigated (Vazey and Aston-Jones, 2012).

## Conclusion

The causal association between dopamine D_2_ receptor agonism and impulse control disorders in Parkinson’s disease has implications for addiction more generally. First, not all individuals develop addictive syndromes following dopamine replacement therapy; those who do appear to have relatively preserved dopamine signaling in the mesolimbic pathway, possibly through a combination of their specific pattern of neurodegeneration, sensitization and pre-morbid vulnerability (as evidenced by the fact that a family history of addiction is a risk factor). It is conceivable that enhanced mesolimbic transmission is also a risk factor in the general population (Buckholtz et al., 2010). Second, it is clear that D_2_ receptor agonism alone is sufficient for the development of the addictive syndrome. While combined D_1_/D_2_ agonists such as L-Dopa may themselves be addictive (Lawrence et al., 2003), D_2_ agonists are not typically administered compulsively; rather, they have the ability to promote other addictions such as pathological gambling (O’Sullivan et al., 2011). This is supported by animal experiments (Collins and Woods, 2009), computational neuroscience models (Cohen and Frank, 2009), and molecular biology evidence (Shen et al., 2008) suggesting that D_1_ receptor stimulation is reinforcing while D_2_ receptor stimulation inhibits the inhibitory indirect pathway. We suggest that D_2_ agonism, in vulnerable individuals, has the effect of “releasing the brake” on reinforcement systems, thus facilitating the development of impulse control disorders. The time-locked nature of the D_2_ effect, and the fact that addictive behaviors typically resolve upon discontinuation of the dopamine agonist, is consistent with the theory that tonic dopamine has an invigorating effect on reward seeking behavior (Niv et al., 2007; Dagher and Robbins, 2009).

We note however that other mechanisms besides dopamine-mediated disruption of responses to reinforcing events and stimuli may play a role. For example, Averbeck et al. (2014) have proposed that Parkinson’s disease patients with impulse control disorders are uncertain about using future information to guide behavior, which could lead to impulsivity (a tendency to privilege immediate action). Also, frontal lobe deficits (Djamshidian et al., 2010) could also lead to impulsivity through impaired self-control. These mechanisms need not be mutually exclusive.

## Conflict of interest statement

The authors declare that the research was conducted in the absence of any commercial or financial relationships that could be construed as a potential conflict of interest.

## References

[B160] Abler B. Walter H. Erk S. Kammerer H. Spitzer M. (2006). Prediction error as a linear function of reward probability is coded in human nucleus accumbens. Neuroimage 31, 790–795 10.1016/j.neuroimage.2006.01.001 16487726

[B1] Albin R. L. Young A. B. Penney J. B. (1989). The functional anatomy of basal ganglia disorders. Trends Neurosci. 12, 366–375 10.1016/0166-2236(89)90074-x 2479133

[B2] Alexander G. E. Crutcher M. D. (1990). Functional architecture of basal ganglia circuits: neural substrates of parallel processing. Trends Neurosci. 13, 266–271 10.1016/0166-2236(90)90107-l 1695401

[B3] Ambermoon P. Carter A. Hall W. D. Dissanayaka N. N. O’Sullivan J. D. (2011). Impulse control disorders in patients with Parkinson’s disease receiving dopamine replacement therapy: evidence and implications for the addictions field. Addiction 106, 283–293 10.1111/j.1360-0443.2010.03218.x 21134016

[B4] American Psychiatric Association (2000). Diagnostic and Statistical Manual of Mental Disorders. 4th Edn., Text Revision, Washington, DC: APA

[B5] Antonini A. Siri C. Santangelo G. Cilia R. Poletti M. Canesi M. (2011). Impulsivity and compulsivity in drug-naive patients with Parkinson’s disease. Mov. Disord. 26, 464–468 10.1002/mds.23501 21312278

[B6] Averbeck B. B. O’Sullivan S. S. Djamshidian A. (2014). Impulsive and compulsive behaviors in Parkinson’s disease. Annu. Rev. Clin. Psychol. 10, 553–580 10.1146/annurev-clinpsy-032813-153705 24313567 PMC4197852

[B7] Bartra O. McGuire J. T. Kable J. W. (2013). The valuation system: a coordinate-based meta-analysis of BOLD fMRI experiments examining neural correlates of subjective value. Neuroimage 76, 412–427 10.1016/j.neuroimage.2013.02.063 23507394 PMC3756836

[B8] Bergh C. Eklund T. Sodersten P. Nordin C. (1997). Altered dopamine function in pathological gambling. Psychol. Med. 27, 473–475 10.1017/s0033291796003789 9089839

[B9] Boileau I. Dagher A. Leyton M. Gunn R. N. Baker G. B. Diksic M. (2006). Modeling sensitization to stimulants in humans: an [11C]raclopride/positron emission tomography study in healthy men. Arch. Gen. Psychiatry 63, 1386–1395 10.1001/archpsyc.63.12.1386 17146013

[B10] Brand M. Labudda K. Kalbe E. Hilker R. Emmans D. Fuchs G. (2004). Decision-making impairments in patients with Parkinson’s disease. Behav. Neurol. 15, 77–85 10.1155/2004/578354 15706051 PMC5488616

[B11] Breiter H. C. Aharon I. Kahneman D. Dale A. Shizgal P. (2001). Functional imaging of neural responses to expectancy and experience of monetary gains and loses. Neuron 30, 619–639 10.1016/s0896-6273(01)00303-8 11395019

[B13] Buckholtz J. W. Treadway M. T. Cowan R. L. Woodward N. D. Li R. Ansari M. S. (2010). Dopaminergic network differences in human impulsivity. Science 329:532 10.1126/science.1185778 20671181 PMC3161413

[B14] Calabresi P. Picconi B. Tozzi A. Di Filippo M. (2007). Dopamine-mediated regulation of corticostriatal synaptic plasticity. Trends Neurosci. 30, 211–219 10.1016/j.tins.2007.03.001 17367873

[B15] Callesen M. B. Scheel-Kruger J. Kringelbach M. L. Moller A. (2013). A systematic review of impulse control disorders in Parkinson’s disease. J. Parkinsons Dis. 3, 105–138 10.3233/JPD-120165 23938342

[B16] Campbell-Meiklejohn D. Wakeley J. Herbert V. Cook J. Scollo P. Ray M. K. (2011). Serotonin and dopamine play complementary roles in gambling to recover losses. Neuropsychopharmacology 36, 402–410 10.1038/npp.2010.170 20980990 PMC3055672

[B17] Campbell-Meiklejohn D. K. Woolrich M. W. Passingham R. E. Rogers R. D. (2008). Knowing when to stop: the brain mechanisms of chasing losses. Biol. Psychiatry 63, 293–300 10.1016/j.biopsych.2007.05.014 17662257

[B18] Canessa N. Crespi C. Motterlini M. Baud-Bovy G. Chierchia G. Pantaleo G. (2013). The functional and structural neural basis of individual differences in loss aversion. J. Neurosci. 33, 14307–14317 10.1523/jneurosci.0497-13.2013 24005284 PMC6618376

[B19] Castellani B. Rugle L. (1995). A comparison of pathological gamblers to alcoholics and cocaine misusers on impulsivity, sensation seeking and craving. Int. J. Addict. 30, 275–289 10.3109/10826089509048726 7790128

[B20] Cavedini P. Riboldi G. Keller R. D’Annucci A. Bellodi L. (2002). Frontal lobe dysfunction in pathological gambling patients. Biol. Psychiatry 51, 334–341 10.1016/s0006-3223(01)01227-6 11958785

[B21] Chang L. J. Sanfey A. G. (2009). Unforgettable ultimatums? Expectation violations promote enhanced social memory following economic bargaining. Front. Behav. Neurosci. 3:36 10.3389/neuro.08.036.2009 19876405 PMC2769546

[B22] Chikama M. McFarland N. R. Amaral D. G. Haber S. N. (1997). Insular cortical projections to functional regions of the striatum correlate with cortical cytoarchitectonic organization in the primate. J. Neurosci. 17, 9686–9705 9391023 10.1523/JNEUROSCI.17-24-09686.1997 PMC6573402

[B23] Christopoulos G. I. Tobler P. N. Bossaerts P. Dolan R. J. Schultz W. (2009). Neural correlates of value, risk, and risk aversion contributing to decision making under risk. J. Neurosci. 29, 12574–12583 10.1523/JNEUROSCI.2614-09.2009 19812332 PMC2794196

[B24] Cilia R. Ko J. H. Cho S. S. van Eimeren T. Marotta G. Pellecchia G. (2010). Reduced dopamine transporter density in the ventral striatum of patients with Parkinson’s disease and pathological gambling. Neurobiol. Dis. 39, 98–104 10.1016/j.nbd.2010.03.013 20338240

[B25] Cilia R. Siri C. Marotta G. Isaias I. U. De Gaspari D. Canesi M. (2008). Functional abnormalities underlying pathological gambling in parkinson disease. Arch. Neurol. 65, 1604–1611 10.1001/archneur.65.12.1604 19064747

[B26] Clark L. Bechara A. Damasio H. Aitken M. R. Sahakian B. J. Robbins T. W. (2008). Differential effects of insular and ventromedial prefrontal cortex lesions on risky decision-making. Brain 131, 1311–1322 10.1093/brain/awn066 18390562 PMC2367692

[B27] Cohen M. X. Frank M. J. (2009). Neurocomputational models of basal ganglia function in learning, memory and choice. Behav. Brain Res. 199, 141–156 10.1016/j.bbr.2008.09.029 18950662 PMC2762323

[B28] Collins G. T. Woods J. H. (2009). Influence of conditioned reinforcement on the response-maintaining effects of quinpirole in rats. Behav. Pharmacol. 20, 492–504 10.1097/fbp.0b013e328330ad9b 19696656 PMC3269766

[B29] Cools R. Lewis S. J. G. Clark L. Barker R. A. Robbins T. W. (2007). L-DOPA disrupts activity in the nucleus accumbens during reversal learning in Parkinson’s disease. Neuropsychopharmacology 32, 180–189 10.1038/sj.npp.1301153 16841074

[B30] Craig A. D. (2002). How do you feel? Interoception: the sense of the physiological condition of the body. Nat. Rev. Neurosci. 3, 655–666 10.1038/nrn894 12154366

[B31] Critchley H. D. Mathias C. J. Dolan R. J. (2001). Neural activity in the human brain relating to uncertainty and arousal during anticipation. Neuron 29, 537–545 10.1016/s1053-8119(01)91735-5 11239442

[B32] Crockford D. N. Goodyear B. Edwards J. Quickfall J. el-Guebaly N. (2005). Cue-Induced brain activity in pathological gamblers. Biol. Psychiatry 58, 787–795 10.1016/j.biopsych.2005.04.037 15993856

[B33] D’Ardenne K. McClure S. M. Nystrom L. E. Cohen J. D. (2008). BOLD responses reflecting dopaminergic signals in the human ventral tegmental area. Science 319, 1264–1267 10.1126/science.1150605 18309087

[B34] Dagher A. Robbins T. W. (2009). Personality, addiction, dopamine: insights from Parkinson’s disease. Neuron 61, 502–510 10.1016/j.neuron.2009.01.031 19249271

[B35] Davie M. (2007). Pathological gambling associated with cabergoline therapy in a patient with a pituitary prolactinoma. J. Neuropsychiatry Clin. Neurosci. 19, 473–474 10.1176/appi.neuropsych.19.4.473 18070857

[B36] De Martino B. Camerer C. F. Adolphs R. (2010). Amygdala damage eliminates monetary loss aversion. Proc. Natl. Acad. Sci. U S A 107, 3788–3792 10.1073/pnas.0910230107 20142490 PMC2840433

[B37] Di Chiara G. Imperato A. (1988). Drugs abused by humans preferentially increase synaptic dopamine concentrations in the mesolimbic system of freely moving rats. Proc. Natl. Acad. Sci. U S A 85, 5274–5278 10.1073/pnas.85.14.5274 2899326 PMC281732

[B38] Djamshidian A. Jha A. O’Sullivan S. S. Silveira-Moriyama L. Jacobson C. Brown P. (2010). Risk and learning in impulsive and nonimpulsive patients with Parkinson’s disease. Mov. Disord. 25, 2203–2210 10.1002/mds.23247 20721918 PMC3093055

[B39] Dodd M. L. Klos K. J. Bower J. H. Geda Y. E. Josephs K. A. Ahlskog J. E. (2005). Pathological gambling caused by drugs used to treat Parkinson disease. Arch. Neurol. 62, 1377–1381 10.1001/archneur.62.9.noc50009 16009751

[B40] Driver-Dunckley E. D. Noble B. N. Hentz J. G. Evidente V. G. Caviness J. N. Parish J. (2007). Gambling and increased sexual desire with dopaminergic medications in restless legs syndrome. Clin. Neuropharmacol. 30, 249–255 10.1097/wnf.0b013e31804c780e 17909302

[B41] Duncan J. Owen A. M. (2000). Common regions of the human frontal lobe recruited by diverse cognitive demands. Trends Neurosci. 23, 475–483 10.1016/s0166-2236(00)01633-7 11006464

[B42] Duvarci I. Varan A. (2000). Descriptive features of Turkish pathological gamblers. Scand. J. Psychol. 41, 253–260 10.1111/1467-9450.00195 11041308

[B43] Eckert M. A. Menon V. Walczak A. Ahlstrom J. Denslow S. Horwitz A. (2009). At the heart of the ventral attention system: the right anterior insula. Hum. Brain Mapp. 30, 2530–2541 10.1002/hbm.20688 19072895 PMC2712290

[B44] Elliott R. Friston K. J. Dolan R. J. (2000). Dissociable neural responses in human reward systems. J. Neurosci. 20, 6159–6165 10934265 10.1523/JNEUROSCI.20-16-06159.2000 PMC6772605

[B45] Evans A. H. Pavese N. Lawrence A. D. Tai Y. F. Appel S. Doder M. (2006). Compulsive drug use linked to senstized ventral striatal dopamine transmission. Ann. Neurol. 59, 852–858 10.1002/ana.20822 16557571

[B46] Falhammar H. Yarker J. Y. (2009). Pathological gambling and hypersexuality in cabergoline-treated prolactinoma. Med. J. Aust. 190, 97 19236300 10.5694/j.1326-5377.2009.tb02289.x

[B47] Frank M. J. O’Reilly R. C. (2006). A mechanistic account of striatal dopamine function in human cognition: psychopharmacological studies with cabergoline and haloperidol. Behav. Neurosci. 120, 497–517 10.1037/0735-7044.120.3.497.supp 16768602

[B48] Frank M. J. Samanta J. Moustafa A. A. Sherman S. J. (2007). Hold your horses: impulsivity, deep brain stimulation and medication in parkinsonism. Science 318, 1309–1312 10.1126/science.1146157 17962524

[B49] Frank M. J. Seeberger L. C. O’Reilly R. C. (2004). By carrot or by stick: cognitive reinforcement learning in parkinsonism. Science 306, 1940–1943 10.1126/science.1102941 15528409

[B50] Frank M. J. (2005). Dynamic dopamine modulation in the basal ganglia: a neurocomputational account of cognitive deficits in medicated and nonmedicated Parkinsonism. J. Cogn. Neurosci. 17, 51–72 10.1162/0898929052880093 15701239

[B51] Frosini D. Pesaresi I. Cosottini M. Belmonte G. Rossi C. Dell’Osso L. (2010). Parkinson’s disease and pathological gambling: results from a functional MRI study. Mov. Disord. 25, 2449–2453 10.1002/mds.23369 20976739

[B52] Gerdeman G. L. Ronesi J. Lovinger D. M. (2002). Postsynaptic endocannabinoid release is critical to long-term depression in the striatum. Nat. Neurosci. 5, 446–451 10.1038/nn832 11976704

[B53] Giovannoni G. O’Sullivan J. D. Turner K. Manson A. J. Lees A. J. (2000). Hedonistic homeostatic dysregulation in patients with Parkinson’s disease on dopamine replacement therapies. J. Neurol. Neurosurg. Psychiatry 68, 423–428 10.1136/jnnp.68.4.423 10727476 PMC1736875

[B54] Goodman A. (2008). Neurobiology of addiction: an integrative review. Biochem. Pharmacol. 75, 266–322 10.1016/j.bcp.2007.07.030 17764663

[B55] Gotham A. M. Brown R. G. Marsden C. D. (1988). ‘Frontal’ cognitive function in patients with Parkinson’s disease ‘on’ and ‘off’ levodopa. Brain 111(Pt. 2), 299–321 10.1093/brain/111.2.299 3378138

[B56] Goudriaan A. E. Oosterlaan J. de Beurs E. van den Brink W. (2005). Decision making in pathological gambling: a comparison between pathological gamblers, alcohol dependents, persons with Tourette syndrome and normal controls. Brain Res. Cogn. Brain Res. 23, 137–151 10.1016/j.cogbrainres.2005.01.017 15795140

[B57] Grace A. A. (2000). The tonic/phasic model of dopamine system regulation and its implications for understanding alcohol and psychostimulant craving. Addiction 95, 119–128 10.1046/j.1360-0443.95.8s2.1.x 11002907

[B58] Grant J. E. Brewer J. A. Potenza M. N. (2006). The neurobiology of substance and behavioural addictions. CNS Spectr. 11, 924–930 17146406 10.1017/s109285290001511x

[B59] Gschwandtner U. Aston J. Renaud S. Fuhr P. (2001). Pathologic gambling in patients with Parkinson’s disease. Clin. Neuropharmacol. 24, 170–172 10.1097/00002826-200105000-00009 11391129

[B60] Hakyemez H. S. Dagher A. Smith S. D. Zald D. H. (2008). Striatal dopamine transmission in healthy humans during a passive monetary reward task. Neuroimage 39, 2058–2065 10.1016/j.neuroimage.2007.10.034 18063390

[B61] Hernandez-Lopez S. Tkatch T. Perez-Garci E. Galarraga E. Bargas J. Hamm H. (2000). D2 dopamine receptors in striatal medium spiny neurons reduce L-type Ca2+ currents and excitability via a novel PLC[beta]1-IP3-calcineurin-signaling cascade. J. Neurosci. 20, 8987–9895 11124974 10.1523/JNEUROSCI.20-24-08987.2000 PMC6773013

[B62] Holman A. (2009). Impulse control disorder behaviors associated with pramipexole used to treat fibromyalgia. J. Gambl. Stud. 25, 425–431 10.1007/s10899-009-9123-2 19241148

[B63] Huettel S. A. Stowe C. J. Gordon E. M. Warner B. T. Platt M. L. (2006). Neural signatures of economic preferences for risk and ambiguity. Neuron 49, 765–775 10.1016/j.neuron.2006.01.024 16504951

[B64] Kable J. W. Glimcher P. W. (2007). The neural correlates of subjective value during intertemporal choice. Nat. Neurosci. 10, 1625–1633 10.1038/nn2007 17982449 PMC2845395

[B65] Kahneman D. Tversky A. (1979). Prospect theory: an analysis of decision under risk. Econometrica 47, 263–291 10.2307/1914185

[B66] Kahnt T. Park S. Q. Cohen M. X. Beck A. Heinz A. Wrase J. (2009). Dorsal striatal-midbrain connectivity in humans predicts how reinforcements are used to guide decisions. J. Cogn. Neurosci. 21, 1332–1345 10.1162/jocn.2009.21092 18752410

[B67] Kish S. J. Shannak K. Hornykiewicz O. (1988). Uneven pattern of dopamine loss in the striatum of patients with idiopathic Parkinson’s disease. Pathophysiologic and clinical implications. N. Engl. J. Med. 318, 876–880 10.1056/nejm198804073181402 3352672

[B68] Knutson B. Adams C. M. Fong G. W. Hommer D. (2001a). Anticipation of increasing monetary reward selectively recruits nucleus accumbens. J. Neurosci. 21:RC159 11459880 10.1523/JNEUROSCI.21-16-j0002.2001 PMC6763187

[B69] Knutson B. Greer S. M. (2008). Anticipatory affect: neural correlates and consequences for choice. Philos. Trans. R. Soc. Lond B Biol. Sci. 363, 3771–3786 10.1098/rstb.2008.0155 18829428 PMC2607363

[B70] Knutson B. Delgado M. R. Phillips P. E. M. (2008). “Representation of subjective value in the striatum,” in Neuroeconomics: Decision Making and the Brain, eds Camerer C. Glimcher P. W. Fehr E. Poldrack R. A. (New York: Academic Press), 398–406

[B71] Knutson B. Fong G. W. Adams C. M. Varner J. L. Hommer D. (2001b). Dissociation of reward anticipation and outcome with event-related fMRI. Neuroreport 12, 3683–3687 10.1097/00001756-200112040-00016 11726774

[B161] Knutson B. Taylor J. Kaufman M. Peterson R. Glover G. (2005). Distributed neural representation of expected value. J. Neurosci. 25, 4806–4812 10.1523/JNEUROSCI.0642-05.2005 15888656 PMC6724773

[B72] Knutson B. Westdorp A. Kaiser E. Hommer D. (2000). FMRI visualization of brain activity during a monetary incentive delay task. Neuroimage 12, 20–27 10.1006/nimg.2000.0593 10875899

[B73] Ko C. H. Liu G. C. Hsiao S. Yen J. Y. Yang M. J. Lin W. C. (2009). Brain activities associated with gaming urge of online gaming addiction. J. Psychiatr. Res. 43, 739–747 10.1016/j.jpsychires.2008.09.012 18996542

[B74] Kreitzer A. C. Malenka R. C. (2007). Endocannabinoid-mediated rescue of striatal LTD and motor deficits in Parkinson’s disease models. Nature 445, 643–647 10.1038/nature05506 17287809

[B75] Kuhnen C. M. Knutson B. (2005). The neural basis of financial risk taking. Neuron 47, 763–770 10.1016/j.neuron.2005.08.008 16129404

[B76] Labudda K. Brand M. Mertens M. Ollech I. Markowitsch H. J. Woermann F. G. (2010). Decision making under risk condition in patients with Parkinson’s disease: a behavioural and fMRI study. Behav. Neurol. 23, 131–143 10.1155/2010/743141 21098967 PMC5434409

[B77] Lawrence A. D. Brooks D. J. Whone A. L. (2013). Ventral striatal dopamine synthesis capacity predicts financial extravagance in Parkinson’s disease. Front. Psychol. 4:90 10.3389/fpsyg.2013.00090 23450713 PMC3583186

[B78] Lawrence A. D. Evans A. H. Lees A. J. (2003). Compulsive use of dopamine replacement therapy in parkinson’s disease: reward systems gone awry? Lancet Neurol. 2, 595–604 10.1016/S1474-4422(03)00529-5 14505581

[B79] Lee J. Y. Seo S. H. Kim Y. K. Yoo H. B. Kim Y. E. Song I. C. (2014). Extrastriatal dopaminergic changes in Parkinson’s disease patients with impulse control disorders. J. Neurol. Neurosurg. Psychiatry 85, 23–30 10.1136/jnnp-2013-305549 24023269

[B80] Litt A. Plassmann H. Shiv B. Rangel A. (2011). Dissociating valuation and saliency signals during decision-making. Cereb. Cortex 21, 95–102 10.1093/cercor/bhq065 20444840

[B81] Lobo D. S. Kennedy J. L. (2006). The genetics of gambling and behavioural addictions. CNS Spectr. 11, 931–939 17146407 10.1017/s1092852900015121

[B82] Loewenstein G. F. Weber E. U. Hsee C. K. Welch N. (2001). Risk as feelings. Psychol. Bull. 127, 267–286 10.1037/0033-2909.127.2.267 11316014

[B83] Mamikonyan E. Siderowf A. D. Duda J. E. Potenza M. N. Horn S. Stern M. B. (2008). Long-term follow-up of impulse control disorders in Parkinson’s disease. Mov. Disord. 23, 75–80 10.1002/mds.21770 17960796 PMC2651355

[B84] Marcellino D. Kehr J. Agnati L. F. Fuxe K. (2012). Increased affinity of dopamine for D(2) -like versus D(1) -like receptors. Relevance for volume transmission in interpreting PET findings. Synapse 66, 196–203 10.1002/syn.21501 22034017

[B85] Menza M. A. Golbe L. I. Cody R. A. Forman N. E. (1993). Dopamine-related personality traits in parkinson’s disease. Neurology 43(Pt. 1), 505–508 10.1212/wnl.43.3_part_1.505 8450991

[B86] Menza M. A. (2000). The personality associated with parkinson’s disease. Curr. Psychiatry Rep. 2, 421–426 10.1007/s11920-000-0027-1 11122991

[B87] Mink J. W. (1996). The basal ganglia: focused selection and inhibition of competing motor programs. Prog. Neurobiol. 50, 381–425 10.1016/s0301-0082(96)00042-1 9004351

[B88] Molina J. A. Sainz-Artiga M. J. Fraile A. Jimenez-Jimenez F. J. Villanueva C. Orti-Pareja M. (2000). Pathologic gambling in Parkinson’s disease: a behavioral manifestation of pharmacologic treatment? Mov. Disord. 15, 869–872 10.1002/1531-8257(200009)15:5<869::aid-mds1016>3.0.co;2-i 11009192

[B89] Montague P. R. Berns G. S. (2002). Neural economics and the biological substrates of valuation. Neuron 36, 265–284 10.1016/s0896-6273(02)00974-1 12383781

[B90] Niv Y. Daw N. D. Joel D. Dayan P. (2007). Tonic dopamine: opportunity costs and the control of response vigor. Psychopharmacology (Berl) 191, 507–520 10.1007/s00213-006-0502-4 17031711

[B91] O’Doherty J. Dayan P. Schultz J. Deichmann R. Friston K. Dolan R. J. (2004). Dissociable roles of ventral and dorsal striatum in instrumental conditioning. Science 304, 452–454 10.1126/science.1094285 15087550

[B92] O’Doherty J. P. Hampton A. Kim H. (2007). Model-Based fMRI and its application to reward learning and decision making. Ann. N Y Acad. Sci. 1104, 35–53 10.1196/annals.1390.022 17416921

[B93] O’Sullivan S. S. Wu K. Politis M. Lawrence A. D. Evans A. H. Bose S. K. (2011). Cue-induced striatal dopamine release in Parkinson’s disease-associated impulsive-compulsive behaviours. Brain 134(Pt. 4), 969–978 10.1093/brain/awr003 21349901

[B94] Ochoa C. Alvarez-Moya E. M. Penelo E. Aymami M. N. Gomez-Pena M. Fernandez-Aranda F. (2013). Decision-making deficits in pathological gambling: the role of executive functions, explicit knowledge and impulsivity in relation to decisions made under ambiguity and risk. Am. J. Addict. 22, 492–499 10.1111/j.1521-0391.2013.12061.x 23952896

[B95] Packard M. G. Knowlton B. J. (2002). Learning and memory functions of the Basal Ganglia. Annu. Rev. Neurosci. 25, 563–593 10.1146/annurev.neuro.25.112701.142937 12052921

[B96] Paulus M. P. Rogalsky C. Simmons A. Feinstein J. S. Stein M. B. (2003). Increased activation in the right insula during risk-taking decision making is related to harm avoidance and neuroticism. Neuroimage 19, 1439–1448 10.1016/s1053-8119(03)00251-9 12948701

[B97] Petry N. M. Stinson F. S. Grant B. F. (2005). Comorbidity of DSM-IV pathological gambling and other psychiatric disorders: results from the National epidemiologic survey on alcohol and related conditions. J. Clin. Psychiatry 66, 564–574 10.4088/jcp.v66n0504 15889941

[B98] Petry N. M. (2001a). Pathological gamblers, with and without substance use disorders, discount delayed rewards at high rates. J. Abnorm. Psychol. 110, 482–487 10.1037//0021-843x.110.3.482 11502091

[B99] Petry N. M. (2001b). Substance abuse, pathological gambling and impulsiveness. Drug Alcohol Depend. 63, 29–38 10.1016/s0376-8716(00)00188-5 11297829

[B100] Pizzagalli D. Evins A. Schetter Erika C. Frank M. J. Pajtas P. Santesso D. (2008). Single dose of a dopamine agonist impairs reinforcement learning in humans: behavioral evidence from a laboratory-based measure of reward responsiveness. Psychopharmacology (Berl) 196, 221–232 10.1007/s00213-007-0957-y 17909750 PMC2268635

[B101] Plassmann H. O’Doherty J. Rangel A. (2007). Orbitofrontal cortex encodes willingness to pay in everyday economic transactions. J. Neurosci. 27, 9984–9988 10.1523/jneurosci.2131-07.2007 17855612 PMC6672655

[B102] Politis M. Loane C. Wu K. O’Sullivan S. S. Woodhead Z. Kiferle L. (2013). Neural response to visual sexual cues in dopamine treatment-linked hypersexuality in Parkinson’s disease. Brain 136(Pt. 2), 400–411 10.1093/brain/aws326 23378222

[B103] Pontone G. Williams J. R. Bassett S. S. Marsh L. (2006). Clinical features associated with impulse control disorders in Parkinson disease. Neurology 67, 1258–1261 10.1212/01.wnl.0000238401.76928.45 17030761

[B104] Potenza M. N. Steinberg M. A. Skudlarski P. Fulbright R. K. Lacadie C. M. Wilber M. K. (2003). Gambling urges in pathological gambling: a functional magnetic resonance imaging study. Arch. Gen. Psychiatry 60, 828–836 10.1001/archpsyc.60.8.828 12912766

[B105] Preuschoff K. Quartz S. R. Bossaerts P. (2008). Human insula activation reflects risk prediction errors as well as risk. J. Neurosci. 28, 2745–2752 10.1523/jneurosci.4286-07.2008 18337404 PMC6670675

[B106] Pritchard T. C. Macaluso D. A. Eslinger P. J. (1999). Taste perception in patients with insular cortex lesions. Behav. Neurosci. 113, 663–671 10.1037//0735-7044.113.4.663 10495075

[B107] Quickfall J. Suchowersky O. (2007). Pathological gambling associated with dopamine agonist use in restless legs syndrome. Parkinsonism Relat. Disord. 13, 535–536 10.1016/j.parkreldis.2006.10.001 17270485

[B108] Rao H. Mamikonyan E. Detre J. A. Siderowf A. D. Stern M. B. Potenza M. N. (2010). Decreased ventral striatal activity with impulse control disorders in Parkinson’s disease. Mov. Disord. 25, 1660–1669 10.1002/mds.23147 20589879 PMC3063061

[B109] Ray N. J. Miyasaki J. M. Zurowski M. Ko J. H. Cho S. S. Pellecchia G. (2012). Extrastriatal dopaminergic abnormalities of DA homeostasis in Parkinson’s patients with medication-induced pathological gambling: a [11C] FLB-457 and PET study. Neurobiol. Dis. 48, 519–525 10.1016/j.nbd.2012.06.021 22766031 PMC3465363

[B110] Reuter J. Raedler T. Rose M. Hand I. Glascher J. Buchel C. (2005). Pathological gambling is linked to reduced activation of the mesolimbic reward system. Nat. Neurosci. 8, 147–148 10.1038/nn1378 15643429

[B111] Reynolds J. N. Hyland B. I. Wickens J. R. (2001). A cellular mechanism of reward-related learning. Nature 413, 67–70 10.1038/35092560 11544526

[B112] Riba J. Krämer U. M. Heldmann M. Richter S. Münte T. F. (2008). Dopamine agonist increases risk taking but blunts reward-related brain activity. PLoS One 3:e2479 10.1371/journal.pone.0002479 18575579 PMC2423613

[B113] Rogers R. D. Lancaster M. Wakeley J. Bhagwagar Z. (2004). Effects of beta-adrenoceptor blockade on components of human decision-making. Psychopharmacology (Berl) 172, 157–164 10.1007/s00213-003-1641-5 14716472

[B162] Rolls E. T. Mccabe C. Redoute J. (2008). Expected value, reward outcome, and temporal difference error representations in a probabilistic decision task. Cereb. Cortex 18, 652–663 10.1093/cercor/bhm097 17586603

[B114] Roy A. Adinoff B. Roehrich L. Lamparski D. Custer R. Lorenz V. (1988). Pathological gambling. A psychobiological study. Arch. Gen. Psychiatry 45, 369–373 10.1001/archpsyc.1988.01800280085011 2451490

[B115] Rutledge R. B. Dean M. Caplin A. Glimcher P. W. (2010). Testing the reward prediction error hypothesis with an axiomatic model. J. Neurosci. 30, 13525–13536 10.1523/jneurosci.1747-10.2010 20926678 PMC2957369

[B116] Sanfey A. G. Rilling J. K. Aronson J. A. Nystrom L. E. Cohen J. D. (2003). The neural basis of economic decision-making in the Ultimatum Game. Science 300, 1755–1758 10.1126/science.1082976 12805551

[B117] Schultz W. Dayan P. Montague P. R. (1997). A neural substrate of prediction and reward. Science 275, 1593–1599 10.1126/science.275.5306.1593 9054347

[B118] Schultz W. Tremblay L. È. Hollerman J. R. (1998). Reward prediction in primate basal ganglia and frontal cortex. Neuropharmacology 37, 421–429 10.1016/s0028-3908(98)00071-9 9704983

[B119] Schultz W. (2002). Getting formal with dopamine and reward. Neuron 36, 241–263 10.1016/s0896-6273(02)00967-4 12383780

[B120] Seedat S. Kesler S. Niehaus D. J. Stein D. J. (2000). Pathological gambling behaviour: emergence secondary to treatment of Parkinson’s disease with dopaminergic agents. Depress. Anxiety 11, 185–186 10.1002/1520-6394(2000)11:4<185::aid-da8>3.3.co;2-8 10945141

[B121] Seeley W. W. Menon V. Schatzberg A. F. Keller J. Glover G. H. Kenna H. (2007). Dissociable intrinsic connectivity networks for salience processing and executive control. J. Neurosci. 27, 2349–2356 10.1523/jneurosci.5587-06.2007 17329432 PMC2680293

[B122] Shaffer H. J. Hall M. N. Vander Bilt J. (1999). Estimating the prevalence of disordered gambling behavior in the United States and Canada: a research synthesis. Am. J. Public Health 89, 1369–1376 10.2105/ajph.89.9.1369 10474555 PMC1508762

[B123] Shen W. Flajolet M. Greengard P. Surmeier D. J. (2008). Dichotomous dopaminergic control of striatal synaptic plasticity. Science 321, 848–851 10.1126/science.1160575 18687967 PMC2833421

[B124] Slutske W. S. Eisen S. True W. R. Lyons M. J. Goldberg J. Tsuang M. (2000). Common genetic vulnerability for pathological gambling and alcohol dependence in men. Arch. Gen. Psychiatry 57, 666–673 10.1001/archpsyc.57.7.666 10891037

[B125] Smeding H. Goudriaan A. Foncke E. Schuurman P. Speelman J. Schmand B. (2007). Pathological gambling after bilateral STN stimulation in Parkinson disease. J. Neurol. Neurosurg. Psychiatry 78, 517–519 10.1136/jnnp.2006.102061 17210626 PMC2117849

[B126] St Onge J. R. Floresco S. B. (2009). Dopaminergic modulation of risk-based decision making. Neuropsychopharmacology 34, 681–697 10.1038/npp.2008.121 18668030

[B127] Steeves T. D. Miyasaki J. Zurowski M. Lang A. E. Pellecchia G. Van Eimeren T. (2009). Increased striatal dopamine release in Parkinsonian patients with pathological gambling: a [11C] raclopride PET study. Brain 132, 1376–1385 10.1093/brain/awp054 19346328 PMC3479148

[B128] Surmeier D. J. Shen W. Day M. Gertler T. Chan S. Tian X. (2010). The role of dopamine in modulating the structure and function of striatal circuits. Prog. Brain Res. 183, 149–167 10.1016/s0079-6123(10)83008-0 20696319 PMC4431764

[B129] Sutton R. S. Barto A. G. (1998). Reinforcement Learning: An Introduction. Cambridge, MA: The MIT Press

[B130] Takahashi H. Fujie S. Camerer C. Arakawa R. Takano H. Kodaka F. (2013). Norepinephrine in the brain is associated with aversion to financial loss. Mol. Psychiatry 18, 3–4 10.1038/mp.2012.7 22349782

[B131] Thut G. Schultz W. Roelcke U. Nienhusmeier M. Missimer J. Maguire R. P. (1997). Activation of the human brain by monetary reward. Neuroreport 8, 1225–1228 10.1097/00001756-199703240-00033 9175118

[B132] Tippmann-Peikert M. Park J. G. Boeve B. F. Shepard J. W. Silber M. H. (2007). Pathologic gambling in patients with restless legs syndrome treated with dopaminergic agonists. Neurology 68, 301–303 10.1212/01.wnl.0000252368.25106.b6 17242339

[B133] Tom S. M. Fox C. R. Trepel C. Poldrack R. A. (2007). The neural basis of loss aversion in decision-making under risk. Science 315, 515–518 10.1126/science.1134239 17255512

[B134] Tricomi E. M. Delgado M. R. Fiez J. A. (2004). Modulation of caudate activity by action contingency. Neuron 41, 281–292 10.1016/s0896-6273(03)00848-1 14741108

[B135] van der Meer M. Kurth-Nelson Z. Redish A. D. (2012). Information processing in decision-making systems. Neuroscientist 18, 342–359 10.1177/1073858411435128 22492194 PMC4428660

[B136] van Eimeren T. Ballanger B. Pellecchia G. Miyasaki J. M. Lang A. E. Strafella A. P. (2009). Dopamine agonists diminish value sensitivity of the orbitofrontal cortex: a trigger for pathological gambling in Parkinson’s disease[quest]. Neuropsychopharmacology 34, 2758–2766 10.1038/sj.npp.npp2009124 19741594 PMC2972251

[B138] Vazey E. M. Aston-Jones G. (2012). The emerging role of norepinephrine in cognitive dysfunctions of Parkinson’s disease. Front. Behav. Neurosci. 6:48 10.3389/fnbeh.2012.00048 22848194 PMC3404393

[B139] Verdejo-Garcia A. Lawrence A. J. Clark L. (2008). Impulsivity as a vulnerability marker for substance-use disorders: review of findings from high-risk research, problem gamblers and genetic association studies. Neurosci. Biobehav. Rev. 32, 777–810 10.1016/j.neubiorev.2007.11.003 18295884

[B140] Vickery T. J. Chun M. M. Lee D. (2011). Ubiquity and specificity of reinforcement signals throughout the human brain. Neuron 72, 166–177 10.1016/j.neuron.2011.08.011 21982377

[B141] Vitaro F. Arseneault L. Tremblay R. E. (1999). Impulsivity predicts problem gambling in low SES adolescent males. Addiction 94, 565–575 10.1046/j.1360-0443.1999.94456511.x 10605852

[B142] Voon V. Gao J. Brezing C. Symmonds M. Ekanayake V. Fernandez H. (2011). Dopamine agonists and risk: impulse control disorders in Parkinson’s; disease. Brain 134(Pt. 5), 1438–1446 10.1093/brain/awr080 21596771 PMC3097893

[B143] Voon V. Pessiglione M. Brezing C. Gallea C. Fernandez H. H. Dolan R. J. (2010). Mechanisms underlying dopamine-mediated reward bias in compulsive behaviors. Neuron 65, 135–142 10.1016/j.neuron.2009.12.027 20152119 PMC2822730

[B144] Voon V. Potenza M. N. Thomsen T. (2007a). Medication-related impulse control and repetitive behaviors in Parkinson’s disease. Curr. Opin. Neurol. 20, 484–492 10.1097/WCO.0b013e32826fbc8f 17620886

[B145] Voon V. Rizos A. Chakravartty R. Mulholland N. Robinson S. Howell N. A. (2014). Impulse control disorders in Parkinson’s disease: decreased striatal dopamine transporter levels. J. Neurol. Neurosurg. Psychiatry 85, 148–152 10.1136/jnnp-2013-305395 23899625 PMC4031642

[B146] Voon V. Thomsen T. Miyasaki J. M. de Souza M. Shafro A. Fox S. H. (2007b). Factors associated with dopaminergic drug-related pathological gambling in Parkinson disease. Arch. Neurol. 64, 212–216 10.1001/archneur.64.2.212 17296836

[B147] Wager T. D. Rilling J. K. Smith E. E. Sokolik A. Casey K. L. Davidson R. J. (2004). Placebo-induced changes in FMRI in the anticipation and experience of pain. Science 303, 1162–1167 10.1126/science.1093065 14976306

[B148] Weintraub D. Koester J. Potenza M. N. Siderowf A. D. Stacy M. Voon V. (2010). Impulse control disorders in Parkinson disease: a cross-sectional study of 3090 patients. Arch. Neurol. 67, 589–595 10.1001/archneurol.2010.65 20457959

[B149] Weintraub D. Siderowf A. D. Potenza M. N. Goveas J. Morales K. H. Duda J. E. (2006). Association of dopamine agonist use with impulse control disorders in Parkinson disease. Arch. Neurol. 63, 969–973 10.1001/archneur.63.7.969 16831966 PMC1761054

[B150] Weller J. A. Levin I. P. Shiv B. Bechara A. (2009). The effects of insula damage on decision-making for risky gains and losses. Soc. Neurosci. 4, 347–358 10.1080/17470910902934400 19466680

[B151] Wexler B. E. Gottschalk C. H. Fulbright R. K. Prohovnik I. Lacadie C. M. Rounsaville B. J. (2001). Functional magnetic resonance imaging of cocaine craving. Am. J. Psychiatry 158, 86–95 10.1176/appi.ajp.158.1.86 11136638

[B152] Wise R. A. Rompre P. P. (1989). Brain dopamine and reward. Annu. Rev. Psychol. 40, 191–225 10.1146/annurev.psych.40.1.191 2648975

[B153] Wise R. A. (1996). Addictive drugs and brain stimulation reward. Annu. Rev. Neurosci. 19, 319–340 10.1146/annurev.neuro.19.1.319 8833446

[B154] Wise R. A. (2013). Dual roles of dopamine in food and drug seeking: the drive-reward paradox. Biol. Psychiatry 73, 819–826 10.1016/j.biopsych.2012.09.001 23044182 PMC3548035

[B155] Wray I. Dickerson M. G. (1981). Cessation of high frequency gambling and withdrawal’ symptoms. Br. J. Addict. 76, 401–405 10.1111/j.1360-0443.1981.tb03238.x 6947814

[B163] Yacubian J. Glascher J. Schroeder K. Sommer T. Braus D. F. Buchel C. (2006). Dissociable systems for gain- and loss-related value predictions and errors of prediction in the human brain. J. Neurosci. 26, 9530–9537 10.1523/JNEUROSCI.2915-06.2006 16971537 PMC6674602

[B156] Yarkoni T. Poldrack R. A. Nichols T. E. Van Essen D. C. Wager T. D. (2011). Large-scale automated synthesis of human functional neuroimaging data. Nat. Methods 8, 665–670 10.1038/nmeth.1635 21706013 PMC3146590

[B157] Ye Z. Hammer A. Camara E. Münte T. F. (2010). Pramipexole modulates the neural network of reward anticipation. Hum. Brain Mapp. 32, 800–811 10.1002/hbm.21067 21484950 PMC6870097

[B158] Zald D. H. Boileau I. El-Dearedy W. Gunn R. McGlone F. Dichter G. S. (2004). Dopamine transmission in the human striatum during monetary reward tasks. J. Neurosci. 24, 4105–4112 10.1523/jneurosci.4643-03.2004 15115805 PMC6729274

[B159] Zuckerman M. Neeb M. (1979). Sensation seeking and psychopathology. Psychiatry Res. 1, 255–264 10.1016/0165-1781(79)90007-6 298353

